# Computational Exploration of Xe Dimers Inside Fullerene Cages

**DOI:** 10.1021/acs.jpca.5c02438

**Published:** 2025-08-08

**Authors:** Athul Santha Bhaskaran, Sílvia Osuna, Marcel Swart

**Affiliations:** † 16738 Institut de Química Computacional i Catàlisi and Departament de Química Universitat de Girona Parc R+i Univ. Girona, Ed. Monturiol, c/ Emili Grahit 91, 17003 Girona, Spain; ‡ ICREA Pg. Lluís Companys 23, 08010 Barcelona, Spain

## Abstract

A systematic analysis for the determination of the optimum fullerene cage for encapsulation of xenon dimers was carried out using density functional theory and activation strain analysis. Our calculations indicate that tubular-like fullerenes are better candidates for the encapsulation of xenon atoms. However, the tubular-like structure should have at least a diameter that is proportional to the van der Waals radius of encapsulated atoms. Our calculations indicate that the smallest fullerene that can stabilize the encapsulation of the xenon dimers in an energetically favorable dimeric state is Xe_2_@C_120_ ([10,0] C_120_-*D*_5*h*_(10766)). When going to higher order fullerenes, the dispersion interaction will dominate over all other interactions. However, the additional space provided by the tubular-like fullerene leads to elongation of the distance between the encapsulated xenon atoms, thus hampering the formation of a xenon–xenon chemical bond.

## Graphical abstract

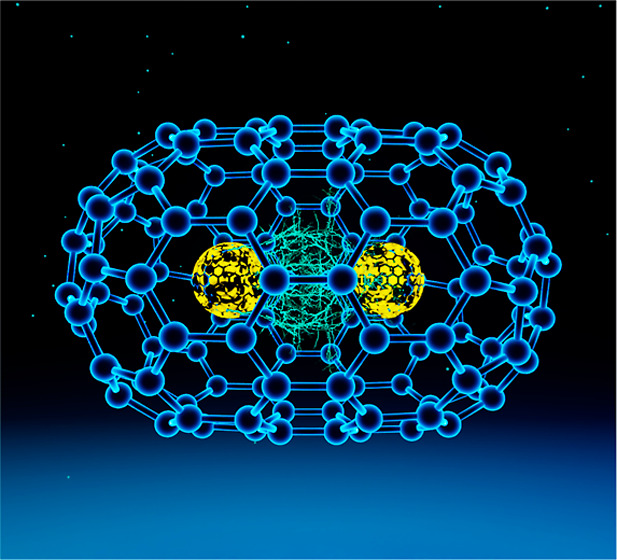

## Introduction

The IUPAC defines a covalent bond as “*a region of relatively high electron density between nuclei which arises at least partly from sharing of electrons and gives rise to an attractive force and characteristic internuclear distance*”. This definition of a chemical bond has, however, sparked controversy in the literature. Some of these issues were addressed by Frenking and Krapp’s in their seminal 2007 paper,^1^ where they discussed the possibility of a Ng–Ng bond inside a C_60_ fullerene. They considered the interaction between noble gas atoms (Ng = Ar, Kr, and Xe) inside the fullerene as a chemical bond and explained the results entirely based on quantum chemical calculations. However, the endohedral Xe_2_@C_60_ fullerene (EF), which was shown to be kinetically stable, is highly unlikely to be synthesized in a laboratory due to the high energy of formation, which is accompanied by a large deformation of the C_60_ from its original empty cage form. The Xe–Xe bonding inside the fullerene cage is an example of confinement-induced bonding. Confinement of foreign molecules inside a molecular vessels can significantly affect reactivity, bonding, and the dynamics of the confined species as well as the vessel itself.^2^ Encapsulating noble gas dimers in various confined spaces have been reported in the literature.^3,4^ Studies show that encapsulating noble gas dimers inside the B–N cages and various other small nanocages can induce a covalent character to the bond between the noble gases.^5,6^ In such systems, the existence of a bond between lighter noble gas elements (He–He) based on Lodwin’s definition of a molecule was demonstrated in the literature.^7,8^

The fascinating field of noble gas reactive chemistry has long been a headache for chemists not only due to its experimental difficulties but also because it has the potential to rewrite the existing bonding and reactivity models. The completely filled shell of orbitals of these elements hinders them from forming a chemical bond with other elements. However, Pauling realized^9^ that the heaviest nonradioactive element in the noble gas family (Xe) can react with fluorine. Later, Bartlett proved his claims by synthesizing XePtF_6_, the first xenon compound, in the laboratory.^10^ In the same year, Hoppe et al.^11^ and Chernick et al.^12^ reported, independently from each other, the synthesis of XeF_2_. Since then, many successful attempts have been made to prepare other molecules involving noble gas atoms. Interestingly, all of those compounds contain a strong electronegative atom. In contrast to that, making a Xe–Xe bond was a major challenge until Seppelt and Drews isolated the Xe_2_Sb_4_F_21_ salt.^13^ They could detect the presence of a Xe_2_^+^ ion with a bond length of 3.0871, which could be considered one of the longest bonds known in the literature. More recently, there have been numerous theoretical and experimental studies on compounds that contain Ng–Ng bonds.^6^ Frenking and co-workers theoretically studied the HXeXe**X** (**X** = F to I) and **Y**XeXe**Y**′ (**Y**,**Y′** = F to Br) compounds and found that these compounds form Xe–Xe bonds.^14,15^ Moreover, theoretical calculations on xenon fluorides suggest that the crystal structure exhibits a Xe–Xe covalent bond at high pressure.^16^ Recently, Ferrari et al. reported spectroscopic evidence of a Xe–Xe bond in an Au–Xe ionic complex.^17^ Their findings were supported by quantum chemical studies of the linear Xe–Au^+^–Xe_2_ isomer and the Xe–Xe bond length at the CCSD­(T) level was found to be 4.097 Å, which is shorter than that of the xenon dimer in the gas phase (4.742 Å),^17^ whereas Frenking and Krapp reported a Xe–Xe bond length of 2.494 Å in the Xe_2_@C_60_ icosahedral isomer of C_60_.^1^ This picture changed when a different isomer of C_60_ was studied. All fullerenes C_*n*_ consist of 12 pentagons and $\left(\frac{n}{2}-10\right)$ hexagons, where *n* ≥ 20 (*n* ≠ 22). Normally, the most stable isomers are those in which the pentagons are separated from each other (the so-called isolated pentagon rule, IPR), which is the case for *I*_*h*_-C_60_. However, exceptions to this rule exist,^18^ with adjacent pentagon pairs (APPs) present, in which case the cages are called non-IPR fullerenes. These non-IPR cages are often seen when (clusters of) atoms are encapsulated inside the cage. Indeed, shortly after the Krapp/Frenking paper, a study on a non-IPR variant of Xe_2_@C_60_ with Xe–Xe distance slightly increased (2.507 Å) showed that this non-IPR EF is more stable than the IPR analogue.^19^ The relatively larger volume of the non-IPR fullerene compared to the IPR isomer and the bonding analysis using energy decomposition analysis (EDA) proved that guest molecules can define the structure of host molecules.^14^

Studies concerning endohedral fullerenes are part of an active field of research. For instance, researchers have carried out ab initio MD simulations to study the encapsulation mechanism of noble gases inside fullerenes.^20^ Sure et al.^21^ explored the size of the smallest fullerene that can encapsulate Ng = He, Ne, and Ar atoms by using density functional theory (DFT). They found that the smallest fullerene C_20_ can accommodate a noble gas atom, but the overall species is only kinetically stable up to a certain extent. They observed that one needs to have a cage size of C_50_ or above to attain thermodynamic stability. From a synthetic point of view, only those with C_*n*_ with *n* ≥ 60 have been reported.^22,23^ Tonner et al.^24^ showed that six is the maximum number of xenon atoms that could fit inside the C_60_ cage before the cage breaks apart. Confinement of foreign atoms inside the fullerene cavity can be achieved by arc discharge experiments,^25^ high energy collision experiments,^26^ and molecular surgery methodology techniques.^27^

Generally speaking, the whole family of fullerenes can be divided into two categories: bucky balls and fullertubes. Theorized in 1992,^28^ fullertubes are compounds that exhibit the behavior of both nanotubes and fullerenes and are proposed to have the best of both worlds. Unlike nanotubes, both ends of fullertubes are capped. Hence, they might act as an ideal vessel for the Xe dimer or any linear molecule to reside inside its cavity. There are reports of successful attempts to prepare fullertubes in the laboratory.^29–31^ Ideally, fullertubes have a capsule-like geometry (see Figure 1). This contrasts with the bucky balls that are more spherical in nature. A fullertube can be theorized as a long carbon nanotube with nanocaps at the ends. Each nanocap should contain exactly 6 pentagons, thereby fulfilling the 12 pentagon criteria for the fullerenes. Nanocaps can be defined in terms of roll up vector (*n*,*m*) of carbon nanotubes.

**Figure 1:**
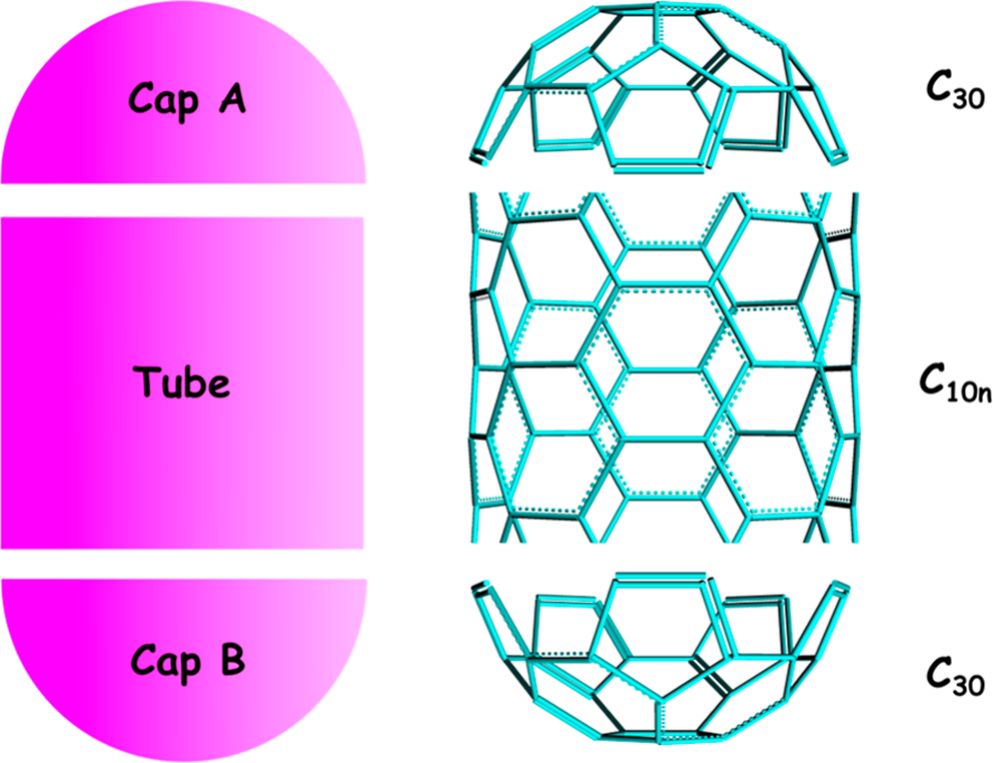
Topology of fullertubes, with an example shown on the right, in which case cap A and cap B consist of 30 carbon atoms (C_30_) and an armchair tube (5,5) with 10 atoms in each ring in the middle. Overall, this leads to the general formula C_30_ + C_30_ + C_10*n*_ for the total number of carbons.

In this work, we explore the presence of xenon dimers inside fullerenes and related structures, continuing the work done by Krapp and Frenking on C_60_. We are extending their work to larger fullerenes and considering the newly synthesized fullertubes. Our goal is to determine the ideal cage-like structure of a fullerene that can support the dimerization of xenon atoms. Also, we are interested in the properties of EFs, in particular, the charge transfer between the cage and the dimer. In the case of Xe_2_@C_60_, Krapp and Frenking reported based on NBO analysis that the Xe_2_ cluster donated ca. 1 electron to the cage, arguably leading to a chemical bond between the xenon atoms.

## Theoretical Method

In this study, we examined the endohedral encapsulation of a Xe_2_ dimer and Xe monomer inside fullerenes using DFT. For the analysis, we have taken fullerenes that are experimentally synthesized in the laboratory as well as the isomers of fullerenes that are known to form endohedral fullerenes. We adapted the fullerene structures from the Fullerene Library,^32^ and some of the structures are generated using the Fullerene program (version 4.5).^33^ The corresponding Xe monomer EF geometries are built by putting the Xe atom at the center of mass of the cage, and Xe dimer EFs are made by placing the xenon dimers along the longest axis at the center; this axis is the one in which the longest diagonal of the 3D convex shape lies (see Scheme S1).

The ADF and QUILD programs^34,35^ were used to optimize the geometries of all species involved, using DFT at the S12g^36^ level with a large TZ2P basis set. Note that S12g incorporates Grimme’s empirical dispersion D3 ^37^ model. The COSMO^38,39^ solvation model was used for solvation effects, with parameters specifically for dichlorobenzene (dielectric constant 9.8, solvent radius 3.54 Å). Relativistic effects are modeled using scalar ZORA Hamiltonian.^40^

## Results and Discussion

Our results on Xe monomer encapsulation in different fullerene cages revealed that the C_70_ cage is the first IPR cage to stabilize the binding of a xenon atom. This is consistent with the experiments by Gadd et al., who were successful in synthesizing the Xe-encapsulated fullerene C_70_ (Xe@C_70_) in the laboratory, which was stable at room temperature for several months.^41^ Moving on to higher fullerenes, the ^133^Xe-encapsulated endohedral fullerenols of C_74_ and C_84_ were also reported as potential candidates for nuclear medicine.^42^ For larger fullerene structures, the binding energy between xenon and the cage saturates at ca. −19 kcal/mol (Figure 2). We found that dispersion interactions between the cage and xenon atoms dominate when we increase the size of the cage. On the other hand, our calculations on the Xe dimer encapsulated inside fullerenes indicate that as we move from C_68_ to C_100_, the overall binding energy of the Xe_2_ dimer inside the cage switches from repulsive to attractive values. This exothermicity might make these higher order EFs synthetically viable in the laboratory. Most importantly, we noted that the Xe–Xe distance in EFs is increasing from 2.765 to 3.422 Å. For comparison based on our DFT calculations, the Xe–Xe distance in the free dimer was found to be 4.445 Å. The stability of the EFs is quantified in terms of binding of the fullerene with Xe_2_ and the binding energy was calculated using eq 1

1 $$Binding\;energy=E_{{Xe}_{2}@C_{2n}}-\left(E_{{Xe}_{2}}+E_{C_{2n}}\right)$$

where $E_{{Xe}_{2}@C_{2n}}$ is the electronic energy of the EF in the solvent, *E*_Xe_2__ electronic energy of the Xe dimer in the solvent, and *E*_C_2*n*__ is the electronic energy of the corresponding empty fullerene in the solvent.

**Figure 2:**
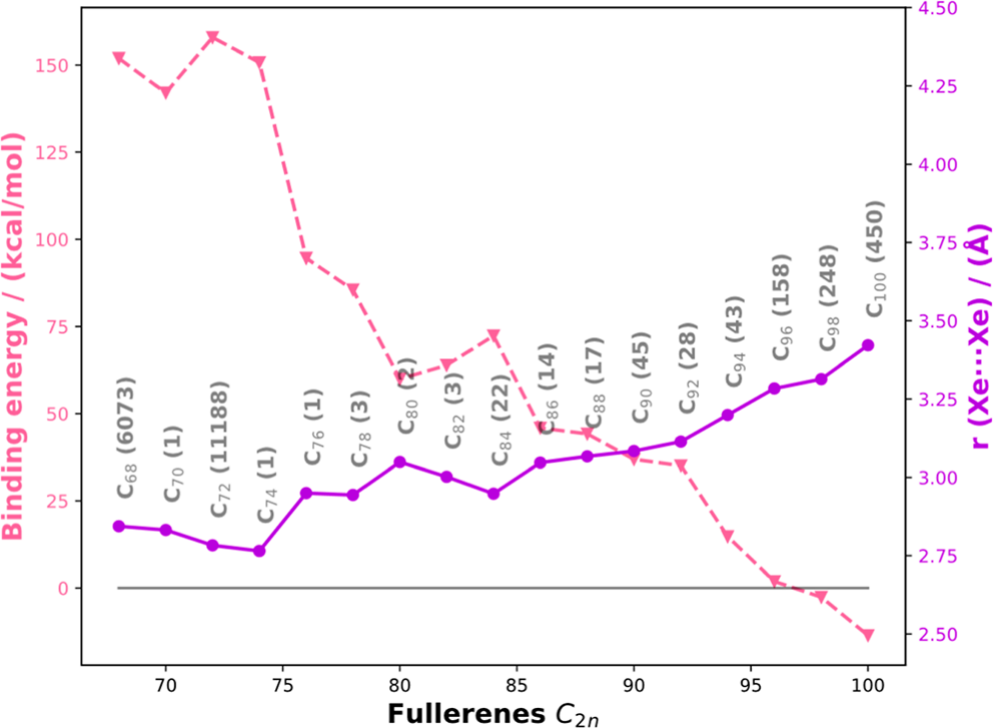
Computed (electronic) binding energies (pink, dotted line) obtained at the S12g-D3/TZ2P level and Xe–Xe distances (purple, solid line) of Xe_2_-encapsulated EF plotted against the number of atoms in the fullerenes. For reference, the optimized distance (S12g-D3/TZ2P) for the Xe–Xe van der Waals dimer is 4.445 Å.

For the non-IPR fullerene family (C_68_), we have considered the structures of experimentally detected endohedral metallofullerenes and found that the isomer 6073 (2 APPs) with a *C*_2*v*_ symmetry is more stable than the other EF isomers considered for the study.^43^ Moving on to higher orders, only one IPR fullerene exists for C_70_, although it can also be considered as a fullertube. So, the tube-like structure of this fullerene provides enough space for the encapsulation of a xenon dimer. However, the EFs of C_68_, C_72_, and C_74_ are less stable than the EF of C_70_ owing to the elongated shape of C_70_. Interestingly, even though an IPR isomer of C_72_ exists, the most stable isomer reported in the literature is actually a non-IPR fullerene (isomer 11188 ^18^); the corresponding EF is the most stable among other isomers. When it comes to C_74_, the triplet electronic state is more stable compared with the singlet state. A sudden increase in the stability of EFs was observed when we moved from C_74_ to C_76_ which is attributed to the increase in the Xe–Xe distance. From C_76_ onward, the binding energy drops further (i.e., it becomes less endothermic), and it flips to negative (attractive) values at the end. So, this trend shows that we would find a stable isomer of fullerene with a negative (i.e., favorable) binding energy, which could host our guest xenon dimer when increasing the fullerene size.

To better understand the bonding picture of these complexes, we have carried out an activation strain analysis (ASM)^44,45^ (see Figure 3a–d) also referred to as the distortion/interaction model followed by an EDA.^51–53^ According to the ASM model, the total binding energy can be divided (eq 2) in terms of interaction and strain (preparation energy), which can then be further divided into subcomponents. The strain energy can be further decomposed into cage deformation and Xe–Xe deformation (from the solvent-phase Xe_2_ dimer). The interaction energy can be expressed as the sum of Pauli repulsion, electrostatic interaction, attractive orbital interactions, dispersion energy, and solvation energy (eq 3).^46^

2 $${\Delta E}_{bind}={\Delta E}_{prep}+{\Delta E}_{\mathrm{int}}$$

3 $${\Delta E}_{\mathrm{int}}={\Delta E}_{Pauli}+{\Delta E}_{elstat}+{\Delta E}_{orbit}+{\Delta E}_{disp}+{\Delta E}_{solv}$$

**Figure 3:**
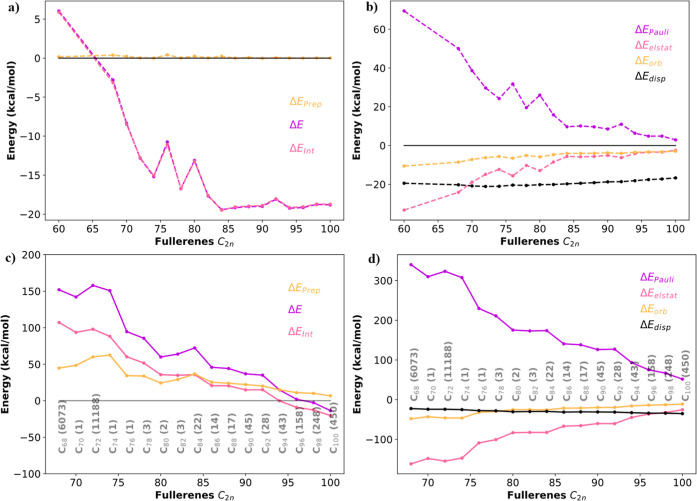
Activation strain analysis and EDA of the interaction energy between fullerenes (Isomer number in the parentheses) and Xe monomer (a,b) and Xe_2_ dimer (c,d). Decomposition of total energy into preparation and interaction (a,c) and of interaction energy into Pauli repulsion, electrostatic interactions, orbital interactions, and dispersion energy (b,d).

In the case of smaller fullerenes, the major component of the strain energy comes from the Xe–Xe deformation, which leads to a large endothermic energy. When we move to higher fullerenes, the Xe_2_ dimer has sufficient space to relax (i.e., it fits inside); as a result, this contribution becomes more and more negligible and starts to tend to zero.

Simultaneously, in the case of smaller fullerenes, Pauli repulsion is the main component of the interaction energy, indicating a repulsive interaction between the xenon dimer and the cages. Later, when the size of the fullerene becomes close to *n* = 98, the interaction energy comes down to the attractive regime. As the number of carbon atoms increases, it results from the attractive dispersion interactions that start to dominate over the repulsive Pauli interactions, thereby stabilizing the overall system. However, this arises from a strengthening of the interaction between Xe atoms and the carbon walls of the fullerene and an increase in the Xe–Xe distance. As expected, the charge transfer between fullerene and the Xe dimer decreases as we move to higher fullerenes.

A multipole-derived charge analysis including monopole and dipole terms (MDC-d) indicates that the degree of charge transfer decreases from 0.933 to 0.443 when we move from C_68_ to C_100_ (see Figure 4). The bond order analysis^47^ using the Gopinathan–Jug (GJ) index (bond orders ≥ 0.001 are considered here) follows the same trend as that of the MDC-d charge analysis. The electrostatic interaction energy based on the discrete polarizable charges on the xenons gives a net repulsion between the xenon atoms (Figure S2).

**Figure 4:**
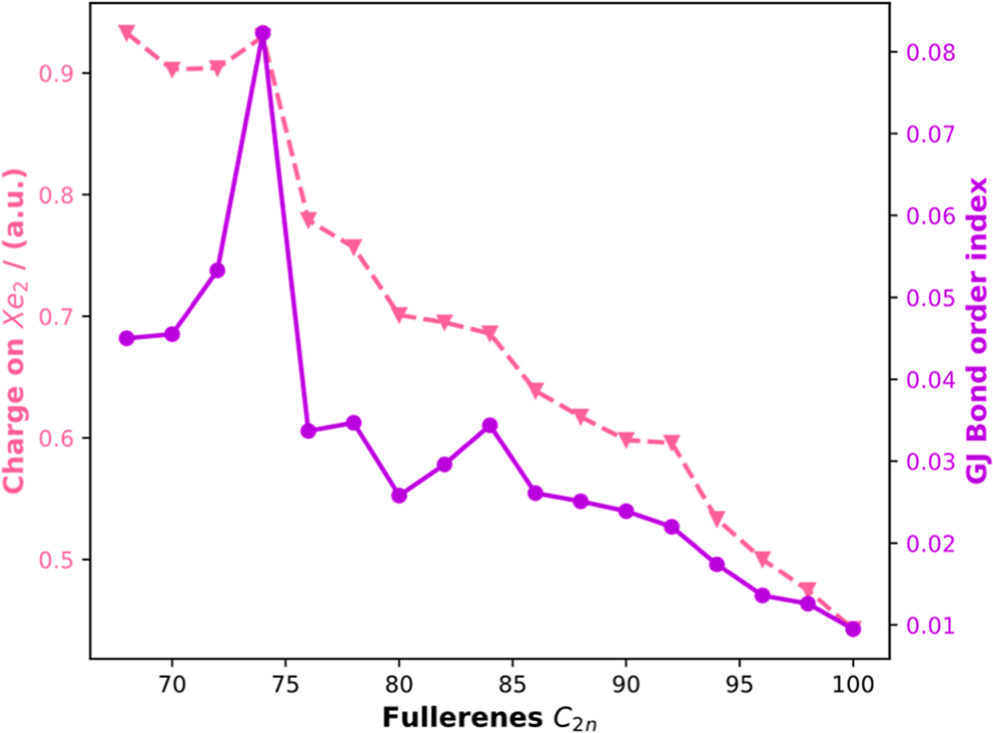
Computed MDC-d charge on Xe_2_ (pinkish, dotted line) and GJ bond order index (BODSEP) (purple, solid line) obtained at the S12g-D3/TZ2P level of Xe_2_-encapsulated EF plotted against the number of atoms in the fullerenes.

Checking the stability of EFs beyond C_100_ is a tedious task as the number of isomers grows as O­(*N* ^9^), where *N* is the number of carbon atoms present in the fullerene cage.^48^ This brings our attention to the recently synthesized novel class of molecules known as fullertubes (vide infra). The capsule-like structure of fullertubes seems to be ideal for the encapsulation of a linear molecule like the Xe_2_ dimer. We have considered different families of fullertubes based on the cap size and the roll-up vector of nanotubes. We consider caps of sizes C_30_, C_36_, C_39_, C_40_, C_42_, C_45_, and C_48_. The corresponding nanotube size was chosen to match the boundary atoms of the caps. The CaGe program was used to generate all nanocap structures.^49^

The topology of a fullertube depends on the rollup vector and the size of the nanocaps attached to it. We have chosen the (*n*,0) and (*n*,*n*) nanotubes for our fullertube generation. Depending on the rollup vector (*n*,*m*), a nanotube can be classified into three groups: (i) *n* = *m* armchair nanotube, (ii) *m* = 0 zigzag nanotube, and (iii) *n* ≠ *m* ≠ 0 chiral nanotubes. Caps boundary can be represented as (23)^*n*^(32)^*m*^,^50,51^ and it should be complementary to the nanotube in the middle.^52^ The simplest cap having 6 pentagons would be a cap with 15 vertices where all pentagons are adjacent to each other, which matches well with a (5,0) nanotube. We have taken IPR caps for our analysis (see Table S3), and the smallest cap we have taken was the half-cut structure of C_60_ consisting of 30 carbon atoms. In principle, we can generate all such hexagon–pentagon patches from specifically slicing IPR fullerenes; however, there are dedicated algorithms available for to do so.^51–53^

We have considered cap A = cap B structures for our analysis, and depending on (*n*,*m*) (see Table 1), we can have various possible hexagon–pentagon patches with exactly six number of isolated pentagons.^51^ Out of all the possibilities listed in Table 1, we chose the IPR caps with a minimum number of carbon atoms for our analysis (see Table S3).

**Table 1:** Number of Possible IPR Caps Linked with the Rollup Vector of the Nanotube (*n*,*m*)

[*n*,*m*]	number of IPR possibilities
5,5	1
9,0	1
10,0	7
6,6	18
11,0	31
12,0	124

We analyzed a total of 7 different fullertube families and their endohedral counterparts. We have used the same binding energy eq (eq 1) as that of spherical fullerenes to calculate the binding energy of endohedrally doped fullertubes. In all the cases, the binding energy reaches a saturated value as the tube length increases. After a certain tube length, the cage framework is no longer pushing the two xenon atoms to form a dimer inside the cage; instead, they promote them to stay as two monomeric species. The van der Waals interaction between the xenons is much weaker than the interactions between the carbons and xenon atoms, which makes the separation of xenon atoms inside the cage much larger than that of the isolated dimeric state. It should be noted that the interaction energy of the xenon dimer with the fullertubes is less than twice the interaction energy of a xenon monomer inside a fullerene. This is not that surprising, given that in the fullerene, the xenon atom is fully surrounded by carbons in the spherical (like) fullerene, yet in the fullertubes, some empty space (of the tube) is surrounding the xenons. This leads to smaller dispersion interactions and hence to a slightly lower interaction energy. Furthermore, we notice that the interaction energy saturates very fast with the length of the tube (see Table S4), which seems to have converged after 2–3 layers of carbon atoms. We also noticed that an increase in the radius of the tubular part increases the binding energy of the fullertubes between xenons and then attains a maximum (negative) value, and after that, it starts to go down (Figure 5, top, and Table 2). From the EDA analysis, it is clear that the Pauli, orbital, and electrostatic interaction are decreasing, whereas the dispersion interaction is dominating as a result of widening the tube radius. Interestingly, we have identified EFs in each fullertube family where the Xe–Xe distance is below the van der Waals distance between xenon dimers, yet they are stabilized by the overall interaction between the cage and xenons.

**Figure 5:**
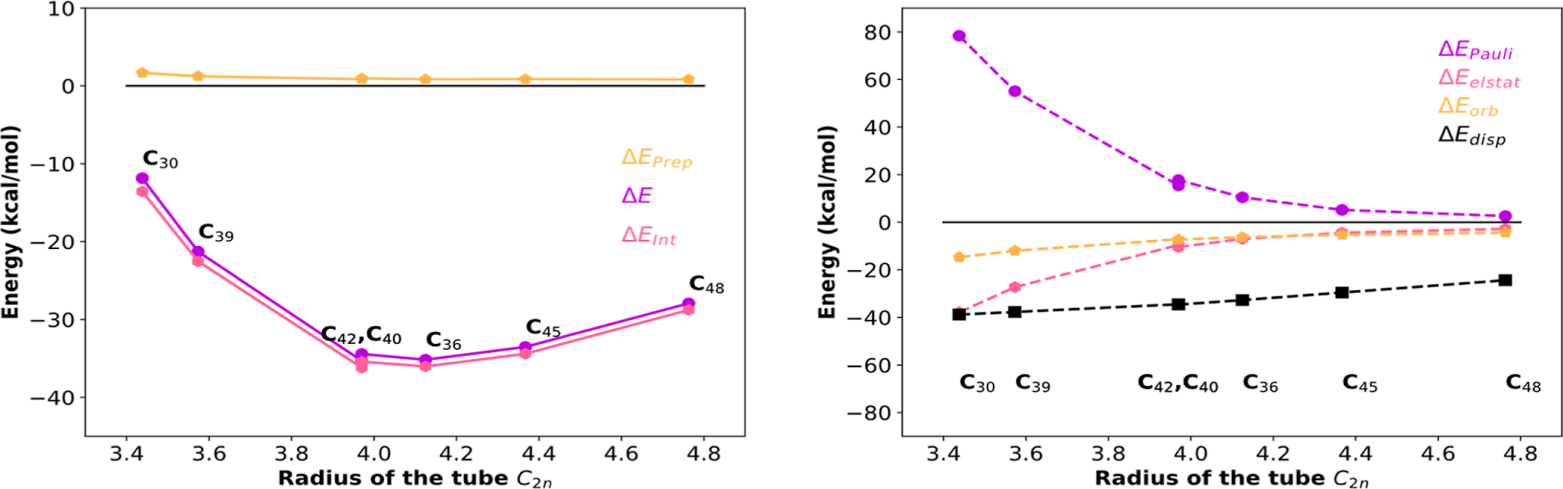
EDA of the interaction energy between the Xe_2_ dimer and the fullertubes. Decomposition of total energy into strain/preparation and interaction (top) and of interaction energy into Pauli repulsion, electrostatic interactions, orbital interactions, and dispersion energy (bottom). Energy values plotted are the saturated value of energy at the different fullertube families plotted against the radius of the fullertube ( $\frac{a\sqrt{3}}{2\pi }\sqrt{n^{2}+m^{2}+nm}$ , *a* = 1.44).

**Table 2:** Most Stable Endohedral Fullertubes with the Xenon Dimer in the Transient Covalent State

fullertubes	[*n*,*m*]	Xe–Xe distance (Å)	charge on dimer Xe_2_ (a.u)	binding energy (kcal/mol)
C_100_	5,5	3.937	0.469	–9.74
C_120_	6,6	3.876	0.280	–31.26
C_114_	9,0	4.247	0.391	–20.84
C_120_	10,0	4.195	0.277	–34.29
C_124_	10,0	4.216	0.280	–33.52
C_134_	11,0	4.116	0.220	–32.77
C_144_	12,0	4.003	0.189	–25.98

Among all the fullertubes, the [10,0] C_120_-*D*_5*h*_ (10766) fullertube in the triplet state (member of the C_40_ + C_40_ + C_20*n*_ fullertube family) would be ideal for the encapsulation of the Xe_2_ dimer in the bonded state. Furthermore, the ASM analysis reveals that the interaction energy contributes most to the binding energy for the [10,0] C_120_-*D*_5*h*_ (10766) isomer, and the deformation energy is almost negligible. Interestingly, the distance between xenons is 4.195 Å, which is shorter than that of the fully relaxed xenon dimer (vide supra). Consequently, there exist some interaction between the xenons. This argument is validated by the charge transfer between the cage and the dimer (Table 2).

The most common topology observed in experimentally synthesized fullertubes corresponds to the armchair pattern of the hexagons. Recently, noble gas-encapsulated endohedral fullerenes have gained more attention among experimentalists, and they have observed the atomic scale time-resolved imaging of Kr_2_@C_120_ species (belonging to the [5,5] fullertube family) in which the covalent state of [Kr_2_]^+^ has been identified.^54^ So, our study on the xenon-encapsulated fullerenes and fullertubes will provide additional insights into the underlying bonding information on noble gas atoms, which may be useful in modeling noble gas clusters inside fullertubes.

## Conclusion

We conducted a systematic study of the xenon dimer- as well as the xenon monomer-encapsulated endohedral fullerene cages computationally using DFT. Our goal was to determine the optimum cage structure that supports the Xe–Xe dimer in a covalently bonded state inside the cavity with an overall stable interaction energy. Our results revealed that as we move from C_68_ to C_100_, the overall binding energy of Xe_2_ inside the cage becomes favorable for binding, thereby making the endohedral fullerenes synthetically viable. The Xe monomer-encapsulated structures on the other hand show a similar trend as that of the dimer ones and previously reported noble gas-encapsulated fullerenes. The main stabilization factor of these EFs is the dispersion interaction between the cage and the xenon atom.

We have also checked the feasibility of encapsulating the Xe_2_ molecule inside a novel class of fullertubes. Our results indicate that the fullertube C_90_ can accommodate the xenon dimer with attractive interactions. However, when exploring larger fuller tubes, the Xe atoms are no longer bonded covalently. Among all the fullertubes tested, the [10,0] C_120_-*D*_5*h*_(10766) isomer shows the most favorable binding energy in the dimeric state. As we move to higher fullertubes, the interactions between the confinement box and the xenons get stronger and reach a maximum value, after which it started to diminish with increasing the Xe–Xe distance.

## Supplementary Material



## Notes

Computed properties of pristine and Xe_2_ endohedral fullerenes; computed properties of pristine and Xe endohedral fullerenes; correlation of Xe_2_ distance with bond order and charge on Xe_2_; correlation of Xe_2_ distance with electrostatic energy between the xenons; correlation between the fullerene length and binding energy of Xe_2_@C_*n*_; correlation between fullerene length and Xe–Xe distance; list of fullertubes studied; computed properties of pristine and endohedral fullertubes; computed triplet-singlet gap of fullertubes; correlation between fullerene volume and Xe–Xe binding energy of fullertubes; EDA-NOCV deformation densities for fullertubes; and minimum distance between xenon atoms and carbons in fullertubes (PDF)

## Abbreviations

C_2*n*_: fullerenes
ZORA: zero-order regular approximation
C_A_ + C_B_ + C_*n*L_: fullertube
