# Supplementary material for: Computational Exploration of Xe Dimers Inside Fullerene Cages
Source: J Phys Chem A. 2025 Aug 8;129(33):7609–16. doi: 10.1021/acs.jpca.5c02438 (PMC12376097; doi:10.1021/acs.jpca.5c02438)
Supplement: Supplementary file 1 [file jp5c02438_si_001.pdf]

Supporting Information for

“Computational exploration of Xe dimers inside fullerene cages”

Athul Santha Bhaskaran,[a] Sílvia Osuna,[a,b]* Marcel Swart[a,b]*

[a] Mr. A. Santha Bhaskaran, Prof. S. Osuna, Prof. M. Swart

Institut de Química Computacional i Catàlisi and Departament de Química

Universitat de Girona

Parc R+i Univ. Girona, Ed. Monturiol, c/ Emili Grahit 91, 17003 Girona, Spain

E-mail: silvia.osuna@icrea.cat, marcel.swart@icrea.cat

[b] Prof. S. Osuna, Prof. M. Swart

ICREA

Pg. Lluís Companys 23, 08010 Barcelona, Spain

Contents

Table S1: Computed properties of pristine and Xe_2 endohedrally doped fullerenes, obtained at S12g-D3/TZ2P (COSMO, ZORA). 3

Table S2: Computed properties of pristine and Xe endohedrally doped fullerenes, obtained at S12g-D3/TZ2P (COSMO, ZORA). 4

Figure S1: Correlation between Xe-Xe distance inside the EMFs and a) GJ bond order index b) Charge on Xe_2 dimer 4

Figure S2: Correlation between Xe-Xe distance inside the EMFs and the electrostatic energy between the xenons. 5

Figure S3: Correlation between the length of fullerene along the major axis and the binding energy between xenon dimer and the cage....6

Figure S4: Correlation between the length of fullerene along the major axis and the Xe-Xe distance of xenon dimer inside the cage....7

Table S3: List of all the fullertube families used for the study and the corresponding caps and the roll up vector. 8

Table S4: ASM and EDA analysis of the Xe_2 doped endohedral fullertubes described in Table S5....10

Table S5: Computed properties of pristine and endohedrally doped fullertubes (singlet electronic state) , obtained at S12g-D3/TZ2P (COSMO, ZORA). 12

Table S6: Computed Triplet -Singlet energy gap of pristine and endohedrally doped fullertubes, obtained at S12g-D3/TZ2P (COSMO, ZORA). 15

Figure S5: Correlation between nuclear volume (V_n) and Xe_2 binding energy of fullertubes....17

Figure S6: EDA-NOCV deformation density plots of fullertube family $C_{30+30+10n}$ (iso value 0.0001). The direction of the charge flow is from red to blue....18

Table S7: Minimum value of C-Xe bond length (Å) in most stable fullertubes in respective fullertube families. 19

References....19

Table S1: Computed properties of pristine and Xe_2 endohedrally doped fullerenes, obtained at S12g-D3/TZ2P (COSMO, ZORA).

Fullerene	MDC-d charge on Xe_2 (a.u.)	Cage deformation energy (kcal/mol)	HOMO-LUMO gap pristine fullerene(eV)	HOMO-LUMO gap of EF (eV)
C_{68} -6073	0.933	6.78	0.31	0.02
C_{70}-1	0.903	9.05	1.74	0.88
C_{72}-11188	0.904	14.65	0.70	0.34
C_{74}-1[a]	0.930	14.97		
C_{76}-1	0.779	6.37	1.08	1.10
C_{78}-3	0.757	5.45	0.87	0.77
C_{80}-2	0.701	3.32	0.46	0.43
C_{82}-3	0.695	4.83	0.73	0.66
C_{84}-22	0.686	8.41	1.04	0.93
C_{86}-14	0.639	4.26	0.22	0.19
C_{88}-17	0.617	4.08	0.66	0.62
C_{90}-45	0.598	3.27	0.76	0.75
C_{92}-28	0.596	2.88	1.33	1.30
C_{94}-43	0.533	1.75	1.12	1.13
C_{96}-158	0.500	0.96	0.60	0.60
C_{98}-248	0.475	0.83	0.89	0.90
C_{100}-450 [a]	0.443	0.32		

[a] species is at triplet state

Table S2: Computed properties of pristine and Xe endohedrally doped fullerenes, obtained at S12g-D3/TZ2P (COSMO, ZORA).

Fullerene	Symmetry	HOMO-LUMO gap pristine fullerene(eV)	HOMO-LUMO gap of EF (eV)	MDC-d charge on Xe (a.u)
C_{60}	Ih(1)	1.68	1.67	0.312683
C_{70}	D5h (1)	1.74	1.74	0.282339
C_{72}	C2V (11188)	0.70	0.70	0.254607
C_{74} [a]	D3h (1)			0.229271
C_{76}	D2(1)	1.08	1.06	0.27899
C_{78}	C2V(3)	0.87	0.76	0.238628
C_{80}	D2(2)	0.46	0.47	0.196656
C_{82}	C2(3)	0.73	0.73	0.211171
C_{84}	D2d(23)	1.10	1.10	0.182394
C_{86}	C2(17)	0.65	0.65	0.151083
C_{88}	Cs(17)	0.66	0.66	0.155317
C_{90}	C2(45)	0.76	0.76	0.154192
C_{92}	D3(28)	1.33	1.33	0.148472
C_{94}	C2(43)	1.12	1.12	0.155825
C_{96}	C2(181)	0.88	0.88	0.133081
C_{98}	C2(248)	0.89	0.88	0.118771
C_{100}	D2(449)	0.41	0.41	0.117555

[a] species is at triplet state

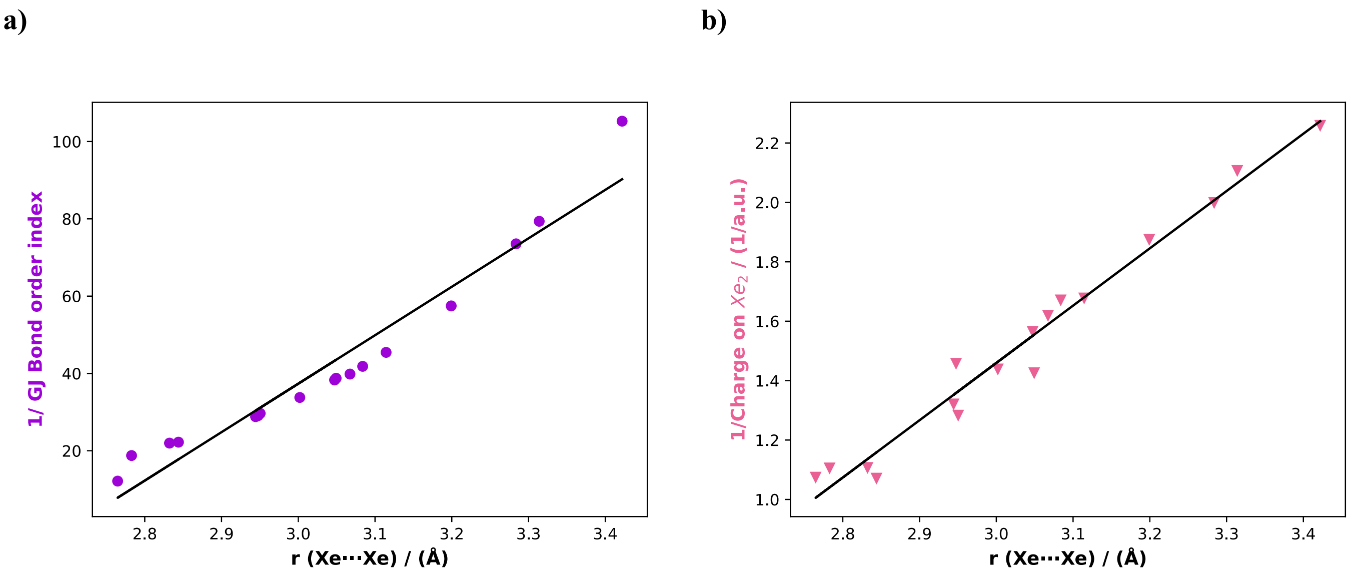

Figure S1: Correlation between Xe-Xe distance inside the EMFs and a) GJ bond order index b) Charge on Xe_2 dimer

Electrostatic energy from the atomic charges (Multipole derived atomic charges)

$$U_{est,ij} = \frac{q_i q_j}{r_{ij}} f_{V,ij}$$

Here the screening factor $f_{v,ij}$ for the potential V accounts for overlapping charge densities.[1]

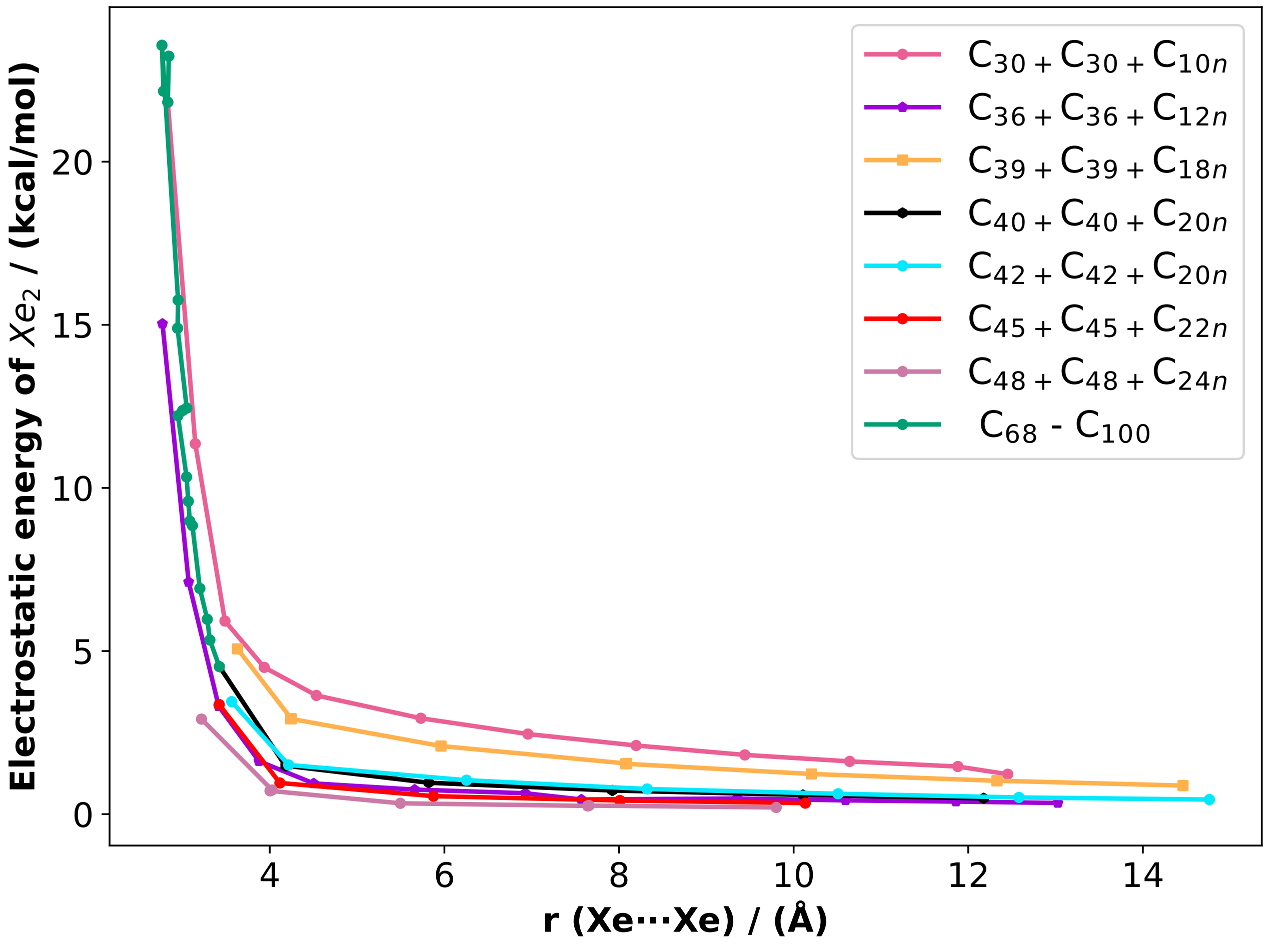

Figure S2: Correlation between Xe-Xe distance inside the EMFs and the electrostatic energy between the xenons.

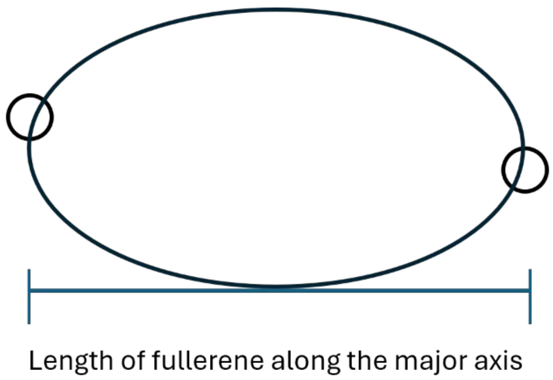

Scheme S1. Length of fullerene along the major axis

It is the distance between the carbon atoms sitting at the extreme ends of the fullerene along one of the Cartesian axis

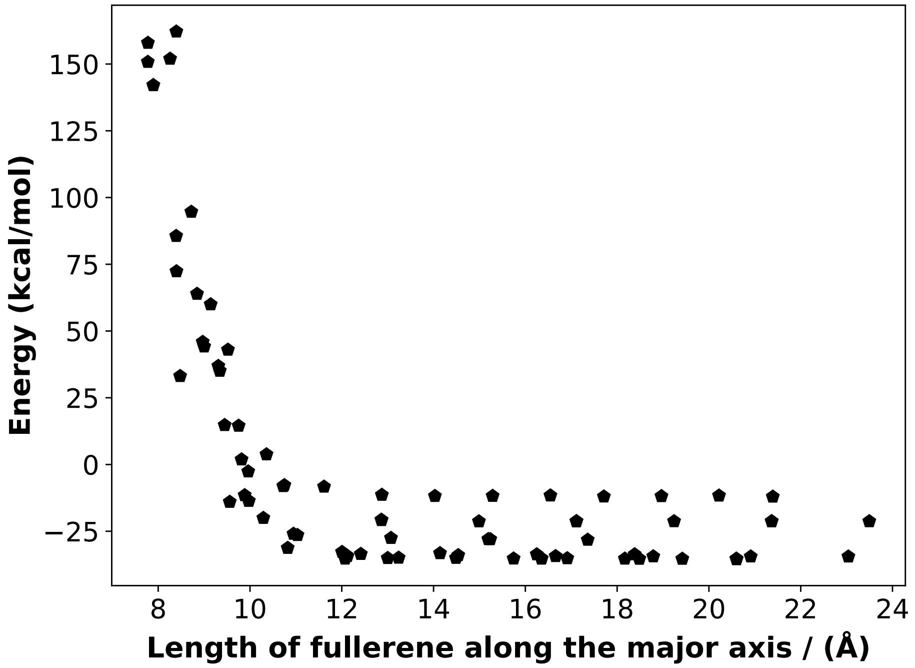

Figure S3: Correlation between the length of fullerene along the major axis and the binding energy between xenon dimer and the cage.

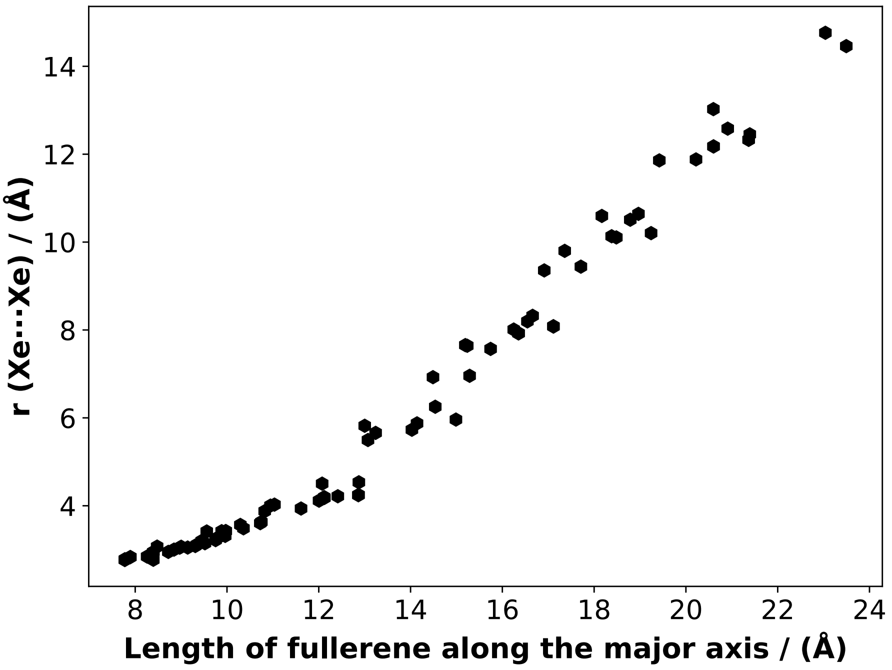

Figure S4: Correlation between the length of fullerene along the major axis and the Xe-Xe distance of xenon dimer inside the cage.

Fullertubes (C_{A+B+L})

A= No. of atoms in Cap A

B= No. of atoms in Cap B

L= No. of atoms in the tube

General Formula C_{A+B+L}

L= l(n+m) for Armchair tubes (l=1,2,3,4,…)

L= 2l(n+m) for Zigzag tubes (l=1,2,3,4,…)

Table S3: List of all the fullertube families used for the study and the corresponding caps and the roll up vector.

Cap	Roll up vector	Number of atoms	Family	Radius(Å)
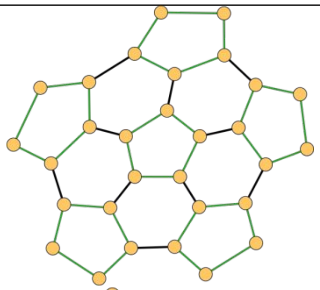	5,5	C_{30}	$C_{30}+C_{30}+C_{10n}$	3.438
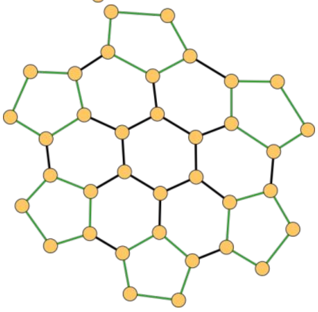	6,6	C_{36}	$C_{36}+C_{36}+C_{12n}$	4.125
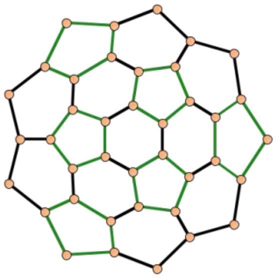	9,0	C_{39}	$C_{39}+C_{39}+C_{18n}$	3.573
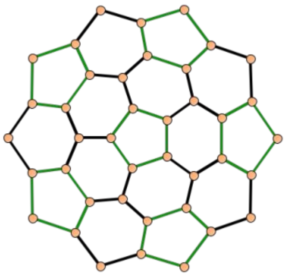	10,0	C_{40}	$C_{40}+C_{40}+C_{20n}$	3.970
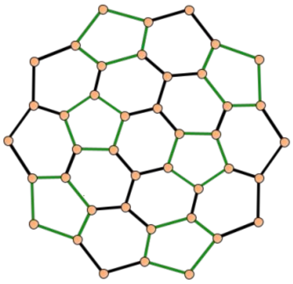	10,0	C_{42}	$C_{42}+C_{42}+C_{20n}$	3.970
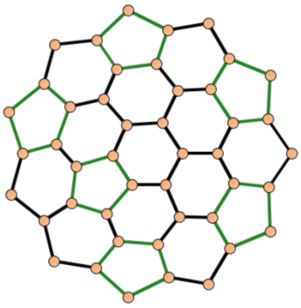	11,0	C_{45}	$C_{45}+C_{45}+C_{22n}$	4.367

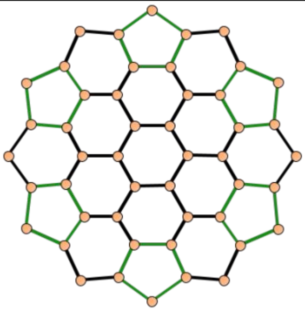	12,0	C_{48}	C_{48}+C_{48}+C_{24n}	4.763

Table S4: ASM and EDA analysis of the Xe_2 doped endohedral fullertubes described in Table S5.

ASM	EDA

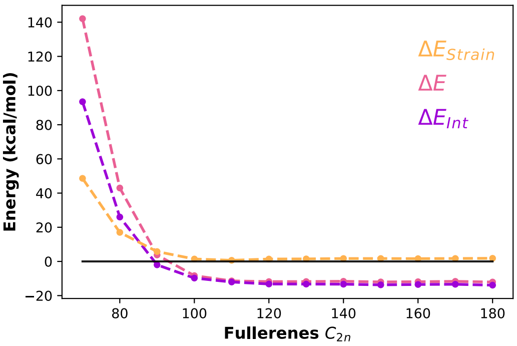

$C_{30+30+10n}$ (5,5)

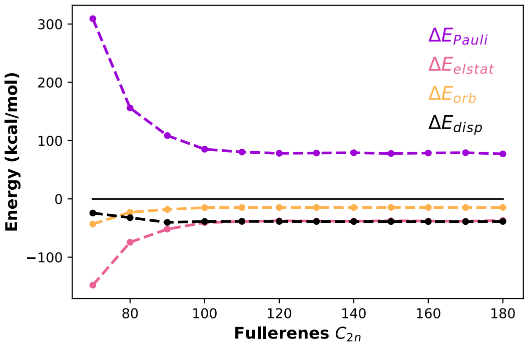

$C_{30+30+10n}$ (5,5)

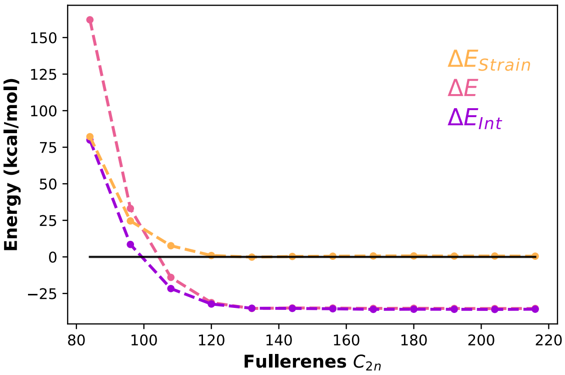

$C_{36+36+12n}$ (6,6)

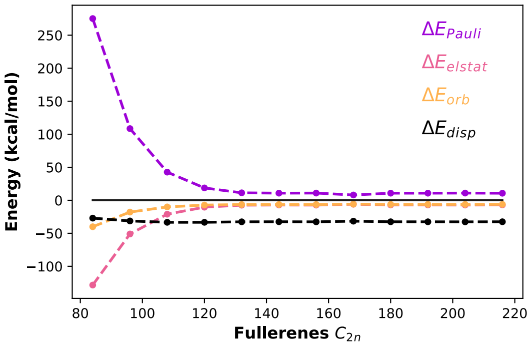

$C_{36+36+12n}$ (6,6)

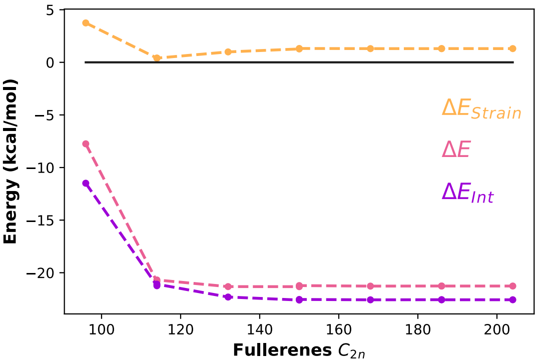

$C_{39+39+18n}$ (9,0)

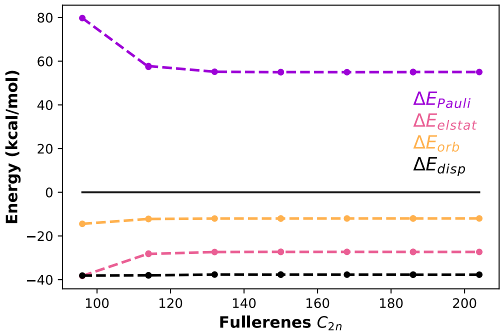

$C_{39+39+18n}$ (9,0)

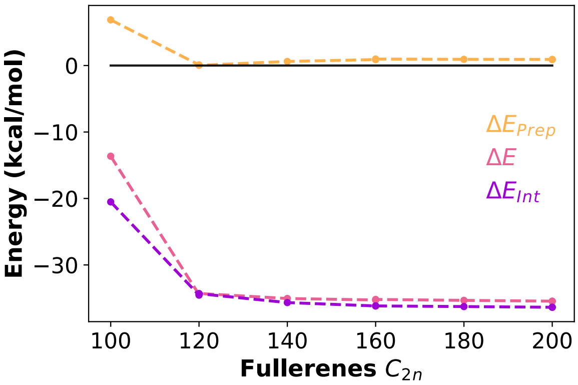

$C_{40+40+20n}$ (10,0)

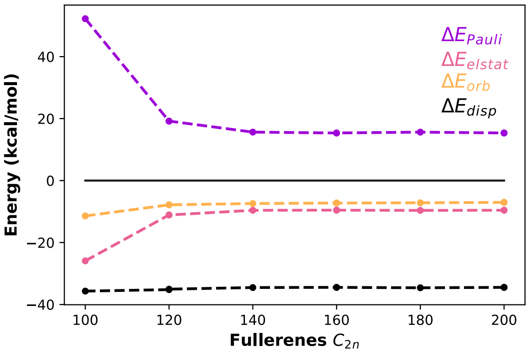

$C_{40+40+20n}$ (10,0)

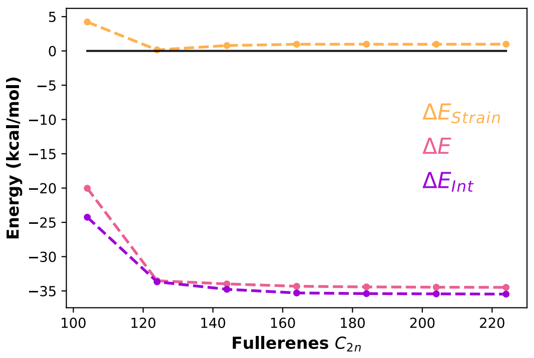

$C_{42+42+20n}$ (10,0)

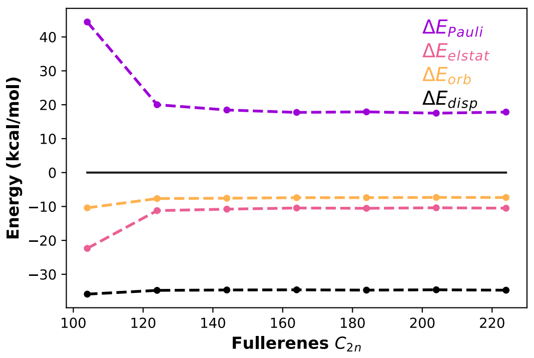

$C_{40+40+20n}$ (10,0)

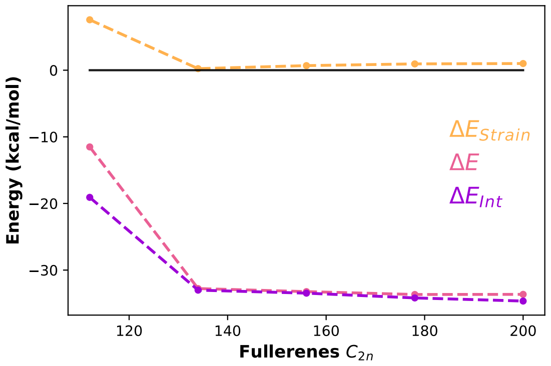

$C_{45+45+22n}$ (11,0)

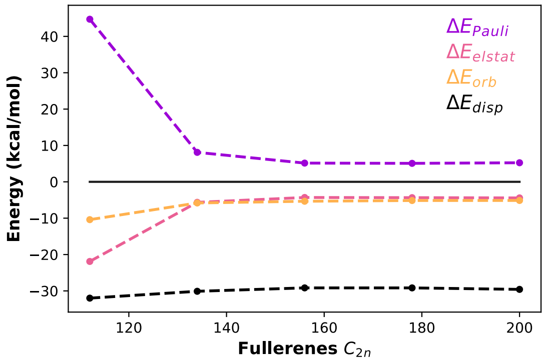

$C_{45+45+22n}$ (11,0)

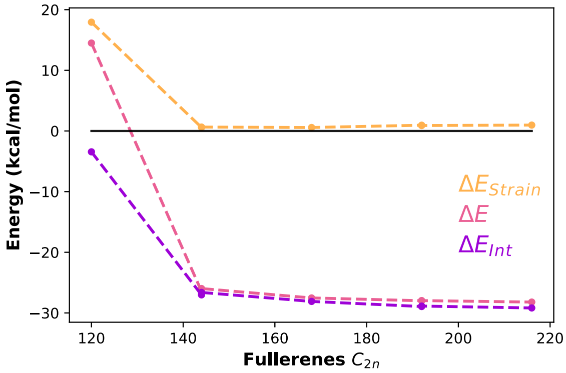

$C_{48+48+24n}$ (12,0)

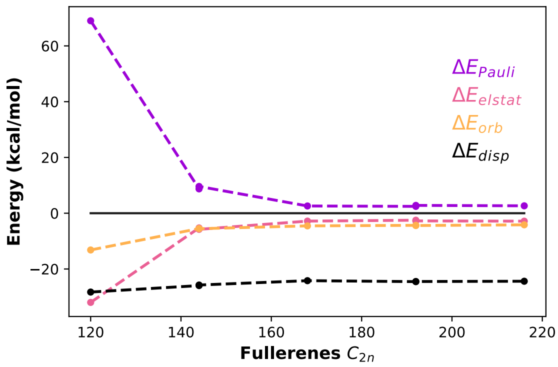

$C_{48+48+24n}$ (12,0)

Table S5: Computed properties of pristine and endohedrally doped fullertubes (singlet electronic state) , obtained at S12g-D3/TZ2P (COSMO, ZORA).

**Fullertube**	**HOMO-LUMO gap EF(eV)**	**HOMO-LUMO gap of pristine fullerene (eV)**	**Cage Deformation energy (kcal/mol)**
C_{70}_30_30_10_D5h	0.884	1.7447	9.054
C_{80}_30_30_20_D5d	0.4114	0.3903	1.211
C_{90}_30_30_30_D5h	1.0566	1.0223	0.454
C_{100}_30_30_40_D5d	1.3895	1.4384	0.624
C_{110}_30_30_50_D5h	0.43	0.4447	0.631
C_{120}_30_30_60_D5d	0.6566	0.645	0.773
C_{130}_30_30_70-D5h	1.112	1.1145	0.742
C_{140}_30_30_80-D5d	0.4232	0.4397	0.786
C_{150}_30_30_90_D5h	0.4428	0.4325	0.773
C_{160}_30_30_100_D5d	0.891	0.8816	0.69
C_{170}_30_30_110_D5h	0.4234	0.4366	0.787
C_{180}_30_30_120-D5d	0.3025	0.2899	0.902
C_{84}-36-36-12	0.263	1.3907	35.261
C_{96}-36-36-24	0.4168	0.1495	4.881
C_{108}-36-36-36	1.0505	1.1039	0.988
C_{120}-36-36-48	1.348	1.3458	0.082
C_{132}-36-36-60	0.3533	0.3445	0.002
C_{144}-36-36-72	0.6978	0.7052	0.016
C_{156}-36-36-84	1.1026	1.096	0.01
C_{168}-36-36-96	0.3353	0.3307	0.022
C_{180}-36-36-108	0.497	0.5051	0.01
C_{192}-36-36-120	0.9242	0.9248	0.02
C_{204}-36-36-132	0.3336	0.3281	0.002
C_{216}-36-36-144	0.3611	0.367	0.008
C_{96}-39-39-18-D3	0.6642	0.6649	0.629

C_{114}-39-39-36-D3h	1.0716	1.0648	0.371
C_{114}-39-39-36-D3	0.5106	0.5108	0.362
C_{132}-39-39-54-D3	0.4697	0.4684	0.353
C_{150}-39-39-72-D3h	0.8073	0.802	0.36
C_{150}-39-39-72-D3	0.3893	0.3883	0.405
C_{168}-39-39-96-D3	0.3618	0.3605	0.375
C_{186}-39-39-108-D3h	0.648	0.6432	0.373
C_{186}-39-39-108-D3	0.3159	0.3154	0.376
C_{204}-39-39-126-D3	0.2947	0.2938	0.374
C_{104}_42-42-20	0.6816	0.6833	0.188
C_{124}_42-42-40	1.0588	1.0547	0.095
C_{144}_42-42-60	0.474	0.473	0.025
C_{164}_42-42-80	0.7638	0.7622	0.056
C_{184}_42-42-100	0.3423	0.342	0.049
C_{204}_42-42-120	0.5521	0.5514	0.04
C_{224}_42-42-140	0.2556	0.2556	0.053
C_{112}-45-45-22	0.3782	0.4064	0.918
C_{134}-45-45-44	0.4684	0.474	0.039
C_{156}-45-45-66	0.228	0.2313	0.042
C_{178}-45-45-88	0.2518	0.2557	0.038
C_{200}-45-45-110	0.1192	0.1224	0.075
C_{120}-48-48-24	0.3234	0.4117	5.2
C_{144}-48-48-48-D6d	0.008	0.0026	0.11
C_{144}-48-48-48-D6h	0.4789	0.4918	0.094
C_{168}-48-48-72-D6	0.2586	0.261	0.086
C_{192}-48-48-96-D6d	0.133	0.1308	0.054
C_{192}-48-48-96-D6h	0.2185	0.2211	0.003
C_{216}-48-48-120-D6	0.0341	0.032	0.044
C_{100}-40-40-20-D5d	0.277	0.0685	0.300

C_{120}-40-40-40-D5d	0.0631	0.0645	0.000
C_{120}-40-40-40-D5h	0.0733	0.0929	0.000
C_{140}-40-40-60-D5d	0.05	0.0505	0.000
C_{160}-40-40-80-D5d	0.046	0.1248	0.000
C_{160}-40-40-80-D5h	0.0467	0.0474	0.100
C_{180}-40-40-100-D5d	0.0444	0.0445	0.000
C_{200}-40-40-120-D5d	0.0493	0.0489	0.000

Table S6: Computed Triplet -Singlet energy gap of pristine and endohedrally doped fullertubes, obtained at S12g-D3/TZ2P (COSMO, ZORA).

Fullertube	Triplet – Singlet Energy gap of Pristine (kcal/mol)	Triplet – Singlet Energy gap of EF (kcal/mol)
C_{84}-36-36-12	28.816	10.561
C_{96}-36-36-24	-0.05	5.859
C_{108}-36-36-36	22.112	20.954
C_{120}-36-36-48	28.821	29.199
C_{132}-36-36-60	5.082	5.309
C_{144}-36-36-72	13.733	13.545
C_{156}-36-36-84	22.908	23.049
C_{168}-36-36-96	5.397	5.526
C_{180}-36-36-108	9.611	9.496
C_{192}-36-36-120	20.484	20.389
C_{204}-36-36-132	5.702	5.812
C_{216}-36-36-144	6.781	6.689
C_{96}-39-39-18-D3	12.38	12.358
C_{114}-39-39-36-D3h	23.233	23.386
C_{114}-39-39-36-D3	9.242	9.228
C_{132}-39-39-54-D3	8.606	8.612
C_{150}-39-39-72-D3h	17.495	17.623
C_{150}-39-39-72-D3	7.024	7.019
C_{168}-39-39-96-D3	6.566	6.577
C_{186}-39-39-108-D3h	14.025	14.147
C_{186}-39-39-108-D3	5.663	5.669
C_{204}-39-39-126-D3	5.312	5.323
C_{100}-40-40-20-D5d	-0.296	-1.063
C_{120}-40-40-40-D5d	-0.185	-0.204
C_{120}-40-40-40-D5h	-0.84	-0.101
C_{140}-40-40-60-D5d	-0.271	-0.278
C_{160}-40-40-80-D5d	-0.83	-0.283
C_{160}-40-40-80-D5h	-0.762	-0.743
C_{180}-40-40-100-D5d	-0.538	-0.533
C_{200}-40-40-120-D5d	-0.827	-0.843
C_{200}-40-40-120-D5h	-0.789	-0.323
C_{104}_42-42-20	13.571	13.576
C_{124}_42-42-40	22.474	22.587
C_{144}_42-42-60	9.279	9.319
C_{164}_42-42-80	16.157	16.207
C_{184}_42-42-100	6.613	6.637
C_{204}_42-42-120	11.577	11.612
C_{224}_42-42-140	4.862	4.878

C_{112}-45-45-22	7.314	6.685
C_{134}-45-45-44	8.915	8.771
C_{156}-45-45-66	3.869	3.799
C_{178}-45-45-88	4.415	4.329
C_{200}-45-45-110	1.657	1.543
C_{120}-48-48-24	7.47	5.233
C_{144}-48-48-48-D6d	-0.762	-0.767
C_{144}-48-48-48-D6h	9.666	9.201
C_{168}-48-48-72-D6	4.714	4.495
C_{192}-48-48-96-D6d	-0.878	-0.885
C_{192}-48-48-96-D6h	3.945	3.765
C_{216}-48-48-120-D6	-0.731	-0.734

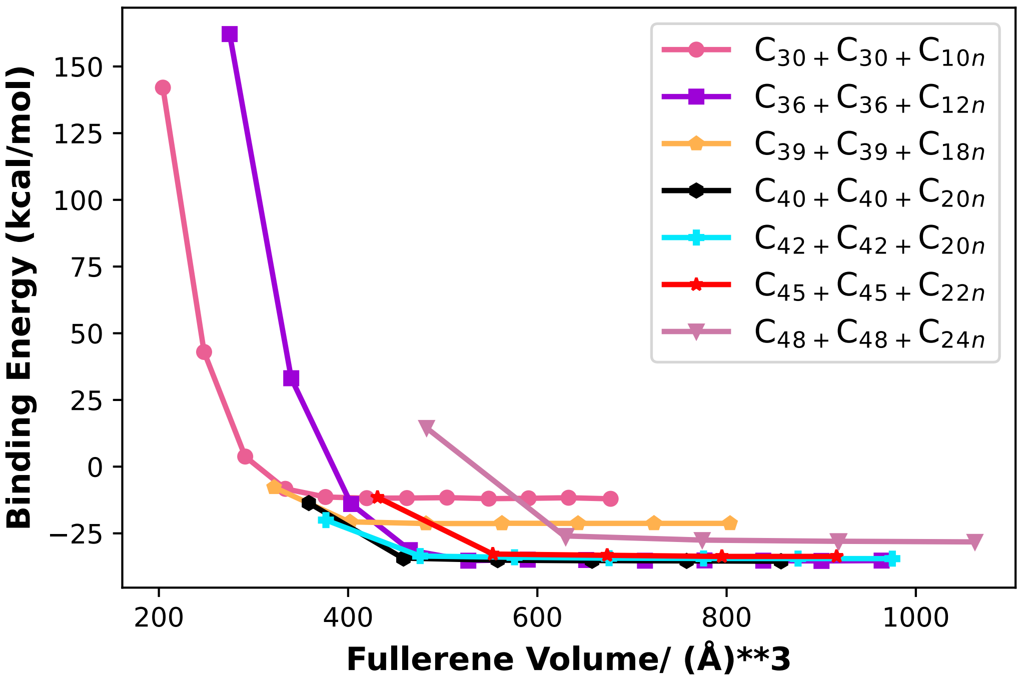

Figure S5: Correlation between nuclear volume (V_n) and Xe_2 binding energy of fullertubes.

Nuclear volume of the fullerenes is defined as

$$Vn(C_{2m}) = \sum_{i}^{12} \sum_{j}^{5} Vp_{ij} + \sum_{i}^{m-10} \sum_{j}^{6} Vh_{ij}$$

We divide the pentagons into 5 triangular faces and hexagons to 6 triangular faces by putting an extra vertex at the center of each faces. Vp and Vh are the volume of tetrahedron created by the center of mass of the fullerene and the triangular faces by assuming fullerenes are convex polyhedras.

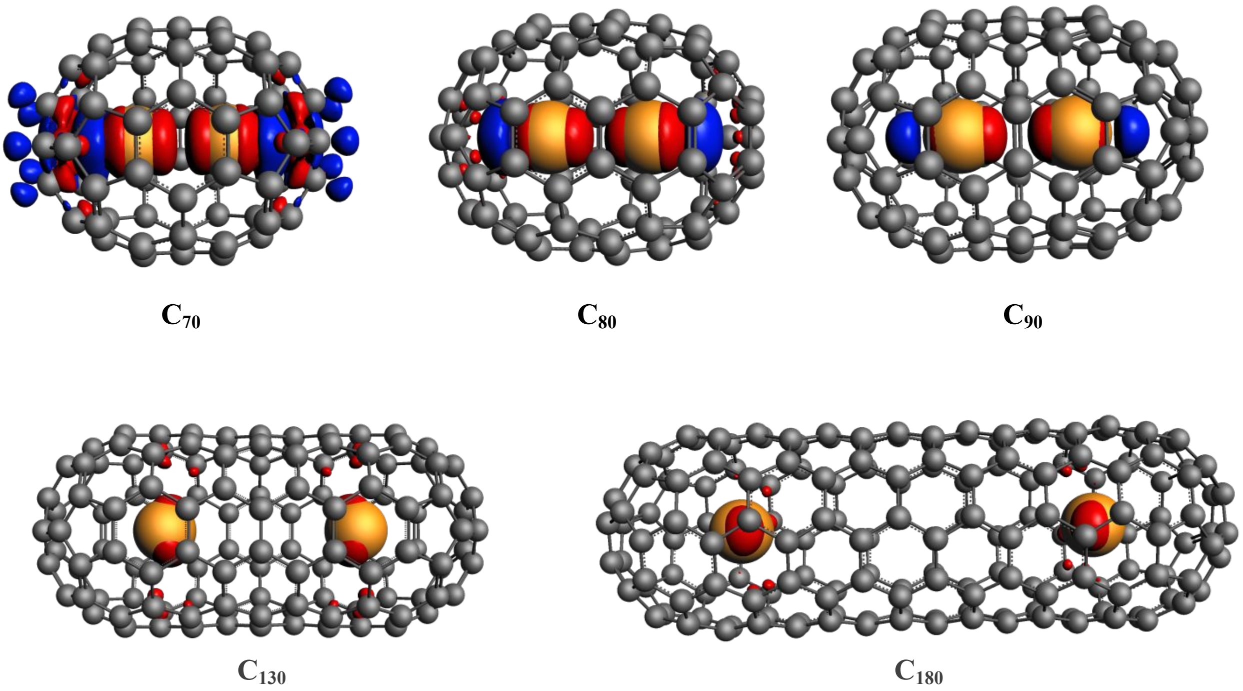

Figure S6: EDA-NOCV deformation density plots of fullertube family $C_{30+30+10n}$ (iso value 0.0001). The direction of the charge flow is from red to blue.

Table S7: Minimum value of C-Xe bond length (Å) in most stable fullertubes in respective fullertube families.

Fullertubes	Minimum C-Xe bond length (Å)
$C_{30}+C_{30}+C_{10n}$	3.51
$C_{36}+C_{36}+C_{12n}$	4.06
$C_{39}+C_{39}+C_{18n}$	3.60
$C_{40}+C_{40}+C_{20n}$	4.00
$C_{42}+C_{42}+C_{20n}$	3.88
$C_{45}+C_{45}+C_{22n}$	4.03
$C_{48}+C_{48}+C_{24n}$	4.06

References

[1] M. Swart, P. Th. Van Duijnen, *Mol. Simul.* 2006, *32*, 471–484.
